# Genetic insights into the timing of metastasis: a secondary analysis of the Count Me In metastatic breast cancer cohort

**DOI:** 10.3389/fcell.2026.1832248

**Published:** 2026-05-29

**Authors:** G. M. Gehling, A. T. Miró-Herrans, J. R. Dungan

**Affiliations:** 1 College of Nursing, University of Florida, Gainesville, FL, United States; 2 College of Nursing, University of Pittsburgh, Pittsburgh, PA, United States; 3 George A. Smathers Library, University of Florida, Gainesville, FL, United States

**Keywords:** *CXCL12*, *GAPDH*, genetic biomarkers, metastatic breast cancer, time to metastasis

## Abstract

**Introduction:**

Metastatic breast cancer is incurable, associated with poor outcomes, and expected to increase in both incidence and cost.

**Methods:**

We conducted a secondary analysis of retrospectively ascertained time-to-event outcomes within a cross-sectional genomic dataset linked to medical and self-reported patient data from the Count Me In: The Metastatic Breast Cancer Project (CMI-MBCProject), available through dbGaP (phs001709.v1.p1). Whole-exome sequencing data derived from saliva samples were bioinformatically processed, and single nucleotide polymorphisms (SNPs) within 50 previously identified metastasis-related genes were selected for analysis. Multivariable-adjusted gamma regression models were used to test associations between SNPs and time to metastasis.

**Results:**

The sample included 63 female participants, with the majority between the ages of 31 and 40 years (n = 29, 46.0%), non-Hispanic (n = 59, 93.7%), and White (n = 55, 87.3%). Time to metastasis was fully observed and right-skewed, with a mean of 732.6 ± 1054.3 days and median of 132.0 days (IQR: 6.0–1018.0). Twenty-three unique genes (58 variants) met the initial screening threshold of p < 0.10. Of these, *CXCL12* rs266091 and *GAPDH* rs1803622 remained significant after false discovery rate correction (q < 0.05), with rs266091 also meeting the more stringent Bonferroni-adjusted threshold. Sensitivity analyses using accelerated failure time Weibull models confirmed the primary findings for both variants.

**Discussion:**

Our analysis identified *CXCL12* and *GAPDH* as promising candidate genes associated with variation in metastatic progression timing. These findings support further investigation into whether genetic risk information could inform targeted surveillance and precision nursing care.

## Introduction

Breast cancer is the most commonly diagnosed cancer among women worldwide. According to a 2022 international report, an estimated 2.3 million new cases occurred, with breast cancer ranking fourth in cancer-related mortality, accounting for approximately 660,000 deaths (Bray et al., 2024). Although screening and prevention strategies have improved, the global burden of breast cancer is expected to increase, with a 38% rise in incidence and a 68% increase in mortality (Kim et al., 2025). Metastatic breast cancer (MBC) occurs when cancer cells spread from the primary breast to regional or distant parts of the body. MBC is associated with poor survival and remains incurable. Gogate et al. (2021) predicted MBC cases would grow from approximately 158,000 in 2015 to over 245,000 in 2030, with a roughly 140% increase in cost (63.4 billion to 152.4 billion, respectively) (Gogate et al., 2021).

Metastatic progression is a complex, multistep biological process involving tumor cell dissemination, dormancy, and colonization of distant organ sites, influenced by both tumor-intrinsic and microenvironmental factors (Valastyan and Weinberg, 2011; Lambert et al., 2017; Massagué and Obenauf, 2016). Prior genomics studies in breast cancer have largely focused on predicting whether metastasis occurs or on survival outcomes (e.g., overall survival) using clinical, molecular, and genomic data (Escala-Garcia et al., 2021; Crawford et al., 2006). Time-to-event modeling approaches, including Cox proportional hazards and parametric survival models, are commonly used in oncology to evaluate disease progression and survival (Aivaliotis et al., 2021). However, relatively few studies have examined the timing of metastatic progression as a continuous outcome in relation to germline genetic variation (Chatrath et al., 2021; Penney et al., 2019).

Among other factors, an individual’s genetic makeup can contribute to their susceptibility to disease development and aggressiveness of disease progression. While hereditary mutations in high-penetrance genes such as *BRCA1* and *BRCA2* account for approximately 5%–10% of all breast cancer cases, the contribution of common germline variants to disease progression and outcomes remains less well understood (Apostolou and Fostira, 2013). Validated biomarkers linked to metastatic development and progression may provide valuable insights into those at increased risk for poor outcomes and toward potential targets for anti-cancer therapeutics.

Candidate genes were selected from our previously published systematic review of single nucleotide polymorphisms (SNPs) associated with metastasis-related outcomes in breast cancer (Gehling et al., 2024). These genes are summarized and grouped by metastasis-related pathways in Table 1. The selected genes span key biological pathways involved in tumor progression and metastatic spread, including immune and chemokine signaling, metabolic regulation, DNA repair, and growth factor response (Witsch et al., 2010; Yin et al., 2022; Hanahan and Weinberg, 2011). For example, chemokine signaling pathways such as *CXCL12*-*CXCR4* are well known to regulate tumor cell migration and organ-specific metastasis, while metabolic pathways influence tumor growth and adaptation under stress conditions (Sun et al., 2014; Zielińska and Katanaev, 2020; Liu et al., 2024). Because the present study used whole-exome sequencing data, two non-coding miRNA genes (miRNA-155 and miRNA-423) were excluded, resulting in a final analytic set of 50 genes. Additional gene-level details, including chromosomal locations, minor allele frequencies, and associated metastasis-related outcomes, are described in the parent systematic review.

**TABLE 1:** Functional classification of candidate genes by metastasis-related pathways.

Pathway category	Candidate genes
Immune regulation and inflammation	*IL2RB, IL10, IL18, IL6, IL7RA, CCL4, CXCL12, CTLA4, TNFSF11, TNFRSF11B, NFKB1, HMGB1, CHI3L1*
Extracellular matrix remodeling and invasion	*MMP2, MMP9, TIMP2, DAAM1*
Cell signaling, growth, and angiogenesis	*ERBB4, VEGFA, KRAS, TGFB1, ESR1, ESR2, VDR, AXIN2, HIF1A, NME1, MAP3K21*
Transcriptional regulation and stress response	*TCF3, ATF3, AHR, ATG16L1, SENP2, ITCH, CASC16*
Metabolism and oxidative stress	*GAPDH, CYP1B1, GALNT16, NR5A2, SOD1, SOD2, TXNRD2, TFAM, BCHE*
DNA repair, cell cycle, and apoptosis	*MGMT, NBN, POLG, CDKN1A, BAX, BBC3*

Candidate genes were derived from a previously published systematic literature review (Gehling et al., 2024) and grouped based on functional annotations from GeneCards and Gene Ontology. Gene symbols reflect official HGNC nomenclature. *TNFSF11* and *TNFRSF11B* encode the proteins commonly referred to as RANKL and OPG, respectively. *CDKN1A* encodes the protein P21. *NBN* is the official symbol for the gene reported as NBS1 in some source studies.

While early-stage breast cancer is often asymptomatic and detected through screening, progression to metastatic disease is associated with substantial clinical burden. Symptoms of metastasis are frequently organ-specific, including bone pain and pathologic fractures, neurologic deficits with brain involvement, dyspnea from pulmonary spread, and constitutional symptoms such as fatigue and weight loss. The methodology of this study is grounded in the Cancer Genomic Integration Model for Symptom Science (CGIMSS), which posits that integrating genomic profiling into symptom research is essential for identifying biological drivers of clinical phenotypes (Grayson et al., 2023). Metastatic progression represents a clinically meaningful transition closely tied to subsequent symptom burden; therefore, identifying genomic predictors of its timing is a critical step within this framework. The objective of this exploratory secondary data analysis was to examine associations between germline SNPs and time to breast cancer metastasis (TtM) within previously identified candidate genes. By situating TtM in the CGIMSS framework, this work aims to establish the biological foundation for future studies that incorporate symptom phenotypes into cancer omics research.

## Materials and methods

### Ethics approval

This retrospective study meets the criteria for nonhuman research due to its use of deidentified data and is exempt from the human consent requirement per the University of Florida (UF) institutional review board (protocol # NH00023882).

### Design

De-identified genomic and clinical data from the Count Me In: The MBC Project (CMI-MBCProject) were retrospectively analyzed as part of a secondary investigation using data accessed through dbGaP (phs001709.v1.p1) and cBioPortal (Wagle et al., 2018). Utilizing a targeted gene association approach on whole-exome deoxyribonucleic acid (DNA), we screened 50 genes previously associated with MBC for variants that may play a role in the timing of metastasis among women diagnosed with MBC.

### Study populations

The CMI-MBCProject was a prospective longitudinal cohort study. Participants from the United States (United States) and Canada (CA) were invited to participate if they had a diagnosis of MBC (i.e., advanced or stage IV). Patients were screened and enrolled online using a virtual consent form. Permission was granted to the study team to access and analyze participants’ medical records and biological samples, including archived formalin-fixed paraffin-embedded (FFPE) tumor tissue, saliva, and blood. Researchers from the parent study used saliva and blood kits to collect germline DNA data and cell-free DNA (cfDNA), respectively. All DNA samples (tumor, germline, and cfDNA) underwent whole exome sequencing (WES).

### Study data sources

#### Genotype

Participant saliva DNA was isolated using the Chemagic Magnetic Separation Module I (chemagic MSM I) in conjunction with the Chemagic DNA Blood Kit-96 (based on PerkinElmer magnetic bead technology), then quantified using a fluorescence-based PicoGreen assay by the CMI MBC study staff. Using a modified version of methods described by Fisher et al., library construction was performed as follows: a reduction from 3μg to 10–100 ng in 50 μL of solution of genomic DNA input, palindromic forked adapters were used in place of Illumina paired end adapters, a reduction of elution volume to 30 μL during the post-enrichment SPRI cleanup, and the addition of a vortexing step. Aside from the palindromic forked adapters, reagents were purchased from KAPA Biosciences in 96-reaction kits. The hybridization and capture step was completed using Illumina’s Nextera Rapid Capture Exome Kit per the manufacturer’s instructions and automated on the Agilent Bravo liquid handling system with the following alterations: 1) pooling of the library construction plate was done before hybridization, and 2) a skirted PCR plate was used in place of the Illumina kit’s Midi plate. To create libraries suitable for cluster amplification and sequencing, library pools were quantified using qPCR via a KAPA biosystems kit and then normalized to 2 nM and denatured using 0.1 N NaOH via the Hamilton Starlet. Per Illumina’s protocol, cluster amplification of denatured templates was completed using HiSeq 4000 cluster chemistry and HiSeq 4000 flow cells, whereby flow cells were sequenced on v1 Sequencing-by-Synthesis chemistry for HiSeq 4000 flow cells. Using RTA v.1.18.64 or later, all flowcells were analyzed, and each pool of whole exome libraries was run on paired 76 bp runs. All exome sequencing had already been completed before the initiation of this secondary data analysis (Wagle et al., 2018).

#### Phenotype

Study covariates were initially collected via medical record extraction and participant self-report survey responses by CMI-MBCProject investigators and staff. All medical record data were reviewed and extracted by a clinical data abstraction specialist based on medical record fields and clinical fields from pathology reports. Whether by imaging or biopsy, the date of primary diagnosis was defined as the earliest confirmation date. Only explicitly reported data in the medical record was obtained by the original study staff; no inferences were made for missing data. Participants’ ages were binned into groups by 5-year intervals by the parent study to maintain patient confidentiality. Survey questions were administered using a free-text response field. To protect patient confidentiality, any racial groups that were listed less than 5 times were changed to ‘other’.

Categorical variables included nine binned age groups established by the CMI-MBCProject parent study to protect patient confidentiality and preserved here for consistency with the source data (“<=30” = 1, “31–35” = 2, “36–40” = 3, “41–45” = 4, “46–50” = 5, “51–55” = 6, “56–60” = 7, “61–65” = 8, “66–70” = 9), as well as each SNP coded at three levels (0 = 0 copies of effect allele, 1 = 1 copy of effect allele, 2 = 2 copies of effect allele). All data were de-identified by the parent study team before being deposited into dbGaP and made available to approved public researchers. The outcome of interest was TtM, previously defined by the parent study as the number of days from initial breast cancer diagnosis (baseline) to the date of metastatic diagnosis (event) based on available medical record data. As all participants must have an MBC diagnosis to be eligible to participate in the study, there were no censored data.

### Bioinformatics approach

**FIGURE 1:**
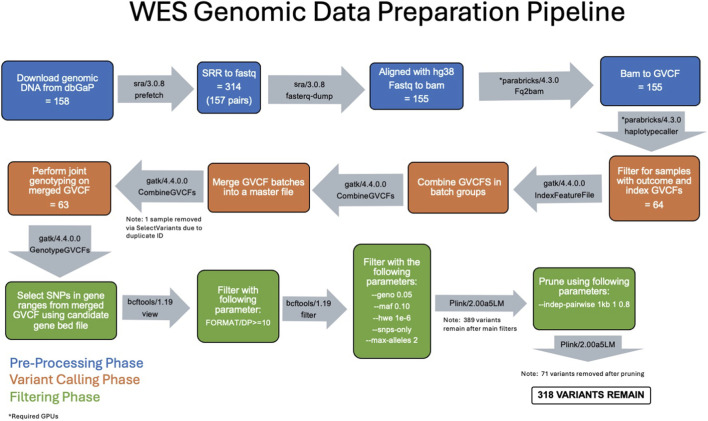
Whole exome sequencing (WES) genomic data preparation pipeline.

Participant data were securely downloaded per dbGaP guidelines once authorized access was granted (https://www.ncbi.nlm.nih.gov/sra/docs/sra-dbgap-download/). The initial cohort made available to researchers in 2019 via a dbGaP repository data dump consisted of 200 individuals; however, only 158 genomic DNA samples were available for retrieval from dbGaP at the time of our study initiation. Some participants contributed more than one sample, but after processing, one sample per participant was retained for analysis. Our bioinformatics approach consisted of three main phases: pre-processing, variant calling, and filtering. A graphical representation of our whole exome sequencing (WES) data processing pipeline can be found in Figure 1.

For the pre-processing phase, we used SRA version 0.0.8 to retrieve genomic DNA files from dbGaP and convert them to FASTQ format. In the variant calling phase, we used GPU-accelerated Parabricks version 4.3.0 to align our FASTQ files to the human reference genome (hg38) and perform genomic variant calling (GVCFs). Genomic Analysis Toolkit (GATK) version 4.4.0.0 was employed to index, combine, and conduct joint genotyping on the merged GVCFs (Van der Auwera et al., 2013). During this phase, we filtered the dataset to include only individuals with available data on our outcome of interest (i.e., TtM in days). We identified a duplicate sample ID (SRR9952524) with conflicting phenotype data, which was subsequently removed, resulting in a sample size of 63 participants, each with one genomic DNA sample.

Using candidate genes identified during our systematic review of the literature, we developed a BED file consisting of 50 genes and their chromosomal locations (Supplementary Table S1). Breast cancer metastasis candidate genes were defined as those that were previously identified among internationally derived gene association studies exploring the relationship between genetic variation and metastasis-related outcomes among individuals diagnosed with breast cancer that were peer reviewed and published within the last 5 years, with a p-value of < 0.05 (Gehling et al., 2024). During the filtering phase, we used BCFtools version 1.19 to select SNPs with a minimum depth of 10x coverage within gene regions based on the developed candidate gene BED file. Additional filtering was performed using PLINK version 2.00a5LM with the following parameters: 5% genotype missingness (--geno 0.05), minor allele frequency (--maf 0.10), Hardy-Weinberg equilibrium (--hwe 1e-6), SNPs-only filtering (--snps-only), and removal of non-biallelic variants (--max-alleles 2). Finally, we used PLINK version 2.00a5LM to filter and remove variants in linkage disequilibrium (--indep-pairwise 1 kb 1 0.8). The resulting SNP dataset consisted of a total of 318 variants available for statistical analysis.

### Statistical analysis

All statistical analyses were performed with RStudio V.2023.12.1 + 402 using the following R packages: BEDMatrix, dplyr, e1071, glm2, survival, and tidyr (Grueneberg and de los Campos, 2019; Therneau, 2026). Genotype data were accessed in binary PLINK format using BEDMatrix to enable memory-efficient analysis of SNP data.

### Model selection

TtM was fully observed for all participants and exhibited right-skewness. Fourteen participants (22%) had same-day documentation of metastasis (TtM = 0 days). Gamma-distributed GLMs provide a flexible framework for modeling skewed, positive continuous outcomes and have been applied across health and behavioral research contexts (Akram et al., 2023; Abadi et al., 2012). For this study, we used a gamma GLM with a log link to estimate multiplicative effects on mean TtM. Because the gamma regression requires strictly positive outcomes, we modeled TtM using a 1-day offset (TtM +1) to accommodate observations with 0 days to metastasis. Exponentiated coefficients from the gamma regression with a log link are interpreted as time ratios (TR), reflecting multiplicative effects on expected TtM. Of note, TR < 1 corresponds to a shorter expected TtM (i.e., earlier metastatic presentation), whereas TR > 1 indicates a longer expected TtM.

### Genotype coding and quality control

SNPs were coded additively based on the effect allele as defined by PLINK (A1 allele), with genotypes modeled as 0, 1, or two copies of the effect allele (Purcell et al., 2007). A minimum minor allele count (MAC >10) threshold was applied to avoid instability due to extremely rare variants. For each SNP, models were fit using participants with non-missing genotypes for that SNP (per-SNP complete-case for genotype). As a result, SNP-specific analytic sample sizes varied slightly (range: 60–63 participants). After quality control, 318 SNPs were evaluated in the primary adjusted analysis.

### Covariate selection and handling of missing data

All regression models were adjusted for age group, race, ethnicity, tumor stage at diagnosis, and ER and HER2 receptor status. These covariates were selected *a priori* based on clinical relevance to metastatic progression and availability across participants. PR status was available in the parent dataset but was not included as a covariate due to near-perfect collinearity with ER status in this sample (Cramér’s V = 0.72; χ^2^ = 65.42, p = 2.1 × 10^−13^), which caused convergence failures in the gamma regression models when both were modeled simultaneously. To preserve a consistent analytic sample across SNP-specific models and minimize data loss, missing values in categorical variables were reclassified as an explicit “UNKNOWN” category. This approach avoids listwise deletion, maintains comparability across models, and accounts for missingness directly within the regression framework rather than implicitly through sample exclusion.

### Primary SNP association analysis

Each SNP was evaluated in separate multivariable-adjusted gamma regression models using an additive genetic model. Major outliers in TtM were retained, as these values were considered to reflect real-world variability in metastatic timing rather than measurement error. Given the modest sample size, SNPs meeting a nominal threshold of p < 0.10 were retained for exploratory screening. To account for multiple testing across SNPs, statistical significance was determined using false discovery rate (FDR) correction, with q < 0.05 considered significant.

### Sensitivity analyses

Accelerated failure time (AFT) models provide an alternative parameterization of event-time data and estimate TRs (also referred to as acceleration factors) representing multiplicative shifts in survival time (Abonongo et al., 2025; Shen et al., 2018). Sensitivity analyses were conducted using adjusted Weibull AFT models for SNPs with q < 0.05 in the primary gamma regression to assess robustness to an alternative event-time framework. AFT models were fit using the same shifted TtM outcome used in gamma models (TtM + 1) to ensure consistent handling of zero-valued times.

A sparsity assessment was performed for top SNPs to evaluate the stability of genetic associations by confirming that MACs were sufficient to support reliable estimation and were not driven by a small number of participants within any single genotype category. To further examine sensitivity to distributional assumptions, additional AFT specifications (log-normal and log-logistic) were evaluated. Wald-based p-values are reported for AFT models, whereas likelihood ratio tests were used for gamma regression models. Direction and magnitude of effects were compared across modeling approaches to evaluate consistency.

## Results

### Demographics

Our sample (N = 63) was entirely female, with the majority of participants between the ages of 31 and 40 (n = 29, 46.0%). Most participants identified as non-Hispanic (n = 59, 93.7%) and White (n = 55, 87.3%). The mean TtM was 732.6 ± 1054.3 days, with a median of 132.0 days (IQR: 6.0–1018.0). Tumors were predominantly estrogen receptor–positive (ER+; n = 53, 84.1%), progesterone receptor–positive (PR+; n = 46, 73.0%), and human epidermal growth factor receptor 2–negative (HER2−; n = 42, 66.7%). Tumor stage at initial breast cancer diagnosis was most commonly stage II (n = 21, 33.3%), followed by stage I (n = 13, 20.6%). Table 2 summarizes the demographic and clinical characteristics of the study cohort.

**TABLE 2:** Participant characteristics (N = 63).

Variable	N (%)
Age	
<=30	4 (6.3%)
31–35	10 (15.9%)
36–40	15 (23.8%)
41–45	8 (12.7%)
46–50	8 (12.7%)
51–55	6 (9.5%)
56–60	8 (12.7%)
61–65	1 (1.6%)
66–70	3 (4.8%)
Race	
White	55 (87.3%)
White, other	3 (4.8%)
Black or african american	2 (3.2%)
Other	2 (3.2%)
Unknown	1 (1.6%)
Ethnicity	
Non-hispanic	59 (93.7%)
Hispanic	3 (4.8%)
Unknown	1 (1.6%)
Tumor stage at diagnosis	
Stage 0	4 (6.3%)
Stage I	13 (20.6%)
Stage II	21 (33.3%)
Stage III	6 (9.5%)
Unknown	19 (30.2%)
ER status	
Negative	8 (12.7%)
Positive	53 (84.1%)
Unknown	2 (3.2%)
HER2 status	
Negative	42 (66.7%)
Positive	19 (30.2%)
Unknown	2 (3.2%)
PR status	
Negative	13 (20.6%)
Positive	46 (73.0%)
Unknown	4 (6.3%)
Time to metastasis (days), mean ± SD; median (IQR)	732.6 ± 1054.3; 132.0 (6.0–1018.0)

Tumor stage at diagnosis was originally recorded using AJCC, substages in the parent study (e.g., IA, IB, IC) and collapsed into major stage categories (0–III) for reporting due to sample size constraints.

### Genetic associations with time to metastasis

Twenty-three unique genes (58 SNPs) met the initial screening threshold (p < 0.10; Supplementary Table S2). Functional annotations were obtained using Ensembl Variant Effect Predictor (VEP; GRCh38) (McLaren et al., 2016). Where multiple annotations were possible due to transcript variability, the most severe consequence was retained. Table 3 presents the two SNPs that remained statistically significant after FDR correction (q < 0.05). Notably, rs266091 remained significant after applying the more stringent Bonferroni correction (p < 0.00015).

**TABLE 3:** Individual SNP associations with time to metastasis using adjusted additive gamma regression models (log link; N = 63).

Gene	SNP	N (genotyped)	MAC	MAF	β (SE)	Mean TR (95% CI)	LRT p-value	BH-FDR
CXCL12**	rs266091	63	16	0.13	−2.2 (0.37)	0.11 (0.05–0.23)	1.0 × 10^−6^	0.0004
*GAPDH*	rs1803622	63	26	0.21	−1.79 (0.35)	0.17 (0.08–0.34)	2.5 × 10^−4^	0.038

Adjusted models include age group, race, ethnicity, tumor stage at diagnosis, ER, status, and HER2 status. TR < 1 indicates accelerated time to metastasis. N represents the number of participants with non-missing genotype data for the SNP.

Abbreviations: MAC, minor allele count; MAF, minor allele frequency; β, log-transformed regression coefficient; TR, time ratio (calculated as exp^β^), multiplicative effect estimate; 95% CI, 95% confidence interval; LRT, likelihood ratio test; BH-FDR, Benjamini–Hochberg false discovery rate.

**Variant also achieved study-wide significance after Bonferroni correction for the number of tested variants (0.05/318 tests; p < 1.57 × 10^−4^).

In multivariable-adjusted gamma regression models, the *CXCL12* variant rs266091 was associated with significantly shorter TtM, with each additional copy of the C effect allele corresponding to an 89% reduction in expected TtM (TR = 0.11, 95% CI: 0.05–0.23; FDR q = 0.0004). The *GAPDH* variant rs1803622 T effect allele was also associated with shorter expected TtM, with each additional copy corresponding to an 83% reduction in expected TtM (TR = 0.17, 95% CI: 0.08–0.34; FDR q = 0.038). Complete multivariable-adjusted gamma results for all SNPs evaluated are provided in Supplementary Table S3.

### Sensitivity analyses

Sensitivity analyses using multivariable-adjusted Weibull AFT models confirmed the primary findings for both rs266091 and rs1803622. Both variants demonstrated strong associations with accelerated TtM (p = 3.1 × 10^−6^ and p = 0.0001, respectively; Table 4). For rs266091, the cohort predominantly carried the C allele: 48 participants (76.2%) were homozygous (carrying two copies) and 14 participants (22.2%) were heterozygous (carrying one copy). Only a single participant (1.6%) carried zero copies of the effect allele. While this reflects a high effect-allele frequency in this sample, the resulting statistical association primarily highlights the contrast between those with one versus two copies of the allele.

**TABLE 4:** Sensitivity analysis of top variants associated with time to metastasis using adjusted Weibull accelerated failure time models (N = 63).

Gene	SNP	β (SE)	z-score	TR (95% CI)	p-value
*CXCL12*	rs266091	−2.15 (0.46)	−4.67	0.12 (0.05–0.29)	3.1 × 10^−6^
*GAPDH*	rs1803622	−1.76 (0.46)	−3.83	0.17 (0.07–0.42)	0.0001

Adjusted models include age group, race, ethnicity, tumor stage at diagnosis, ER, status, and HER2 status. TR < 1 indicates accelerated time to metastasis. These covariates match those utilized in the primary adjusted gamma regression analysis. Negative β coefficients and TR < 1 represent a reduction in time to metastasis, indicating accelerated progression.

Abbreviations: β, log-transformed regression coefficient; z-score, Wald statistic; TR, time ratio (calculated as exp^β^), multiplicative effect estimate; 95% CI, 95% confidence interval.

In contrast, rs1803622 followed a more typical genotype distribution: 41 participants (65.1%) were in the reference group (zero copies of the T allele), 18 (28.6%) were in the heterozygous group (one copy), and 4 (6.3%) were in the homozygous group (two copies). Despite the small size of the homozygous group, the clinical trend remained consistent across genotype categories, with each additional copy of the T allele associated with shorter expected TtM.

Additional sensitivity analyses using alternative accelerated failure time distributions (log-normal and log-logistic) yielded consistent effect directions and statistical significance. These results can be found in Supplementary Table S4.

## Discussion

We screened 50 candidate genes for allelic contributions to timing from diagnosis to metastasis among 63 individuals with MBC from the United States and Canada using a targeted candidate gene approach informed by our previous literature review findings. Of the 23 unique genes (58 SNPs) that met our initial screening threshold of p < 0.10, two SNPs (*CXCL12* rs266091 and *GAPDH* rs1803622) remained statistically significant after FDR correction (q < 0.05). *CXCL12* rs266091 also remained significant after the more stringent Bonferroni correction (p = 1.57 × 10^−4^). These results were consistent in both effect directions and statistical significance across multiple AFT distributions (Weibull, log-normal, log-logistic), supporting the internal consistency of findings across modeling frameworks.

The *CXCL12* pathway is responsible for several essential cellular functions, including immune system monitoring and response, cellular homeostasis, and cancer proliferation and metastatic spread (Yang et al., 2023). Dysregulation in the *CXCL12* signaling pathway has been shown to contribute to the transformation of normal fibroblasts into cancer-associated fibroblasts (CAFs), thereby facilitating cancer cell migration via tumor vascularization and dissemination (Ma et al., 2025; Jia et al., 2025). In contrast to normal fibroblasts, which suppress tumor development, CAFs promote inflammatory reactions, thereby creating a favorable microenvironment and contributing to chemotherapy resistance (Kazakova et al., 2024; Ping et al., 2021). *CXCL12* rs266091 is annotated in dbSNP as a genic downstream variant, a region that may harbor regulatory elements influencing transcriptional activity or 3′ end processing. While the functional consequences of this variant have yet to be characterized, its downstream genomic position warrants investigation into potential regulatory effects on *CXCL12* expression. *CXCL12* signaling is a widely recognized key player in the metastatic spread of breast cancer (Sun et al., 2014; Zielińska and Katanaev, 2020; Wu et al., 2022; Hayasaka et al., 2022). Supporting a role for *CXCL12* in breast cancer susceptibility, a recent meta-analysis revealed Caucasian *CXCL12* rs1801157 homozygous carriers were at increased risk of breast cancer (AA v. GG, OR = 1.35, p = 0.02) (Alijanpour et al., 2024). Extending these findings, we observed an association between rs266091 and a more aggressive expected TtM phenotype among homozygous carriers of the C allele (TR = 0.11; p = 1.0 × 10^−6^). Together, these results suggest that *CXCL12* variants may influence not only breast cancer susceptibility but also disease progression. Further investigation of rs266091 is needed to clarify its functional relevance and evaluate its potential utility as a clinical biomarker for metastatic risk.

*GAPDH* is a key enzyme in glycolysis but is increasingly recognized for additional roles in cellular migration, stress response, and post-transcriptional regulation (Tristan et al., 2011; Garcin, 2019). Although commonly used as a housekeeping gene due to its consistent expression across cell types, accumulating evidence suggests *GAPDH* also contributes to cancer-related metabolic programming and non-glycolytic processes relevant to tumor proliferation (Zhang et al., 2015; Wang et al., 2023). Prior work identified rs1803622, a 3′-UTR variant in *GAPDH* located within a microRNA binding site, as associated with breast cancer risk, implicating altered post-transcriptional regulation of *GAPDH* expression in disease biology (Naghiyan et al., 2020). Our findings extend this literature by demonstrating that rs1803622 is also associated with a more aggressive metastatic phenotype (TR = 0.17; p = 2.5 × 10^−4^). The Warburg effect describes the tendency of cancer cells to favor glycolysis over oxidative phosphorylation, even under oxygen-rich conditions, supporting rapid proliferation and adaptation to metabolic stress (Liberti and Locasale, 2016). Genetic variation affecting *GAPDH* expression or regulation may therefore influence multiple processes relevant to tumor progression. In this context, rs1803622 may contribute to metastatic behavior by modulating *GAPDH*-dependent metabolic and regulatory pathways. Although *GAPDH* has been explored as a metabolic target in preclinical models, clinically established FDA-approved selective inhibitors remain unavailable (Liberti et al., 2017; Ganapathy-Kanniappan et al., 2013).

These results provide a hypothesis-generating foundation for future precision oncology research examining whether carriers of higher-risk genotypes may benefit from earlier or more frequent symptom-focused assessments. Because metastatic progression is often first detected through changes in patient-reported or clinically observed symptoms, genomic markers associated with metastatic timing may offer a biologically informed framework for risk-stratified surveillance. If validated, genotype-stratified surveillance would not replace standard oncology monitoring but could inform the timing and intensity of symptom assessment, patient education, and anticipatory guidance in survivorship care planning.

Importantly, this dataset did not include longitudinal symptom data; therefore, links between genomic biomarkers and symptom trajectories can only be indirectly inferred. The present model supports a conceptual association that requires prospective validation and highlights the importance of incorporating longitudinal symptom phenotyping into future clinical biomarker studies to clarify how these biological signals translate into patient-reported and clinically observed symptom experiences. Such work could examine whether specific genetic profiles are associated with earlier onset, greater severity, or distinct clustering of metastatic symptoms and whether genomic risk modifies not only TtM but also the lived symptom experience of progression.

The role of the oncology nurse in proactive surveillance remains a cornerstone of high-quality metastatic care regardless of genotype (Gennari et al., 2021). Targeted monitoring for early symptoms of common metastatic sites, such as respiratory changes and localized bone pain, is well-established in oncology nursing practice (Shewbridge et al., 2024). If replicated in larger, more diverse cohorts, variants such as *CXCL12* rs266091 and *GAPDH* rs1803622 may help inform whether genotype-stratified surveillance approaches warrant further investigation. Given the exploratory nature of the present findings and the distributional constraints of this sample, particularly for *CXCL12* rs266091, clinical application is premature.

Our study is not without its limitations. First, the modest sample size reduced statistical power to detect true genetic associations and increased the risk of both false negatives and false positives. To mitigate this risk in an exploratory setting, we applied an FDR q < 0.05 and evaluated model stability through extensive sensitivity analyses, demonstrating consistent findings across gamma, Weibull, log-normal, and log-logistic AFT models. Moreover, all participants had a confirmed diagnosis of MBC. As such, TtM was fully observed and variation in TtM may reflect not only underlying biological aggressiveness but also differences in surveillance intensity, diagnostic timing, and access to care. While this outcome captures clinically meaningful heterogeneity in metastatic presentation, it should be interpreted within the context of retrospective event ascertainment rather than as a purely prospective measure of metastatic progression. Furthermore, limited racial and ethnic diversity constrains the broader applicability of our findings. This sample, composed predominantly of young, White, and non-Hispanic women, limits inference for populations that experience disproportionate symptom burden and structural barriers to metastatic care. A more equitable research agenda would prioritize diverse recruitment and explicitly examine whether genomic predictors of metastatic timing operate similarly across racial, ethnic, and socioeconomic groups. Without such representation, precision approaches risk reinforcing existing disparities in symptom recognition and care delivery. In addition, the secondary nature of the dataset precluded adjustment for potentially relevant environmental and lifestyle factors that may intersect with genetic variation to influence breast cancer development and metastatic potential (Gearhart-Serna et al., 2020; Ratajczak-Pawło et al., 2025; Borowsky et al., 2025). These limitations underscore the continued need to increase diverse representation in genomic studies of cancer metastasis. Given the modest sample size and the low frequency of some homozygous genotype groups, particularly for *CXCL12* rs266091, these findings should not be interpreted as population-level effect estimates. Effect sizes may be inflated due to limited sample size and winner’s curse bias, and replication in larger, independent cohorts is required prior to clinical translation. Nonetheless, the consistency of effect direction and statistical significance across multiple modeling frameworks supports the internal consistency of these associations. These findings should be viewed as exploratory, biologically plausible, hypothesis-generating signals regarding metastatic timing, rather than definitive estimates of genetic risk or progression rates. Future studies should prioritize replication of these associations in larger, more diverse, independent cohorts using complementary genotyping approaches, as well as functional characterization of rs266091 and rs1803622 to identify the mechanisms underlying their association with metastatic progression. Integration of germline variation with matched tumor-derived molecular data, including somatic mutation profiles and gene expression, would help clarify how germline variants may contribute to differences in metastatic timing.

## Conclusion

Our analysis identified two promising variants within established candidate genes, *CXCL12* rs266091 and *GAPDH* rs1803622, associated with metastatic timing. These findings demonstrate the feasibility of integrating candidate gene screening with TtM modeling within a precision nursing framework. Within the CGIMSS framework, this work represents an initial step toward incorporating genomic predictors of metastatic timing into symptom science models. By linking genomic variation to metastatic timing, this work provides a biological basis for future research examining how genetic risk may interface with longitudinal symptom trajectories to inform targeted surveillance and risk stratification. Validation in larger and more diverse cohorts will be essential to clarify molecular drivers of metastatic timing and support equitable, personalized nursing care.

## Data Availability

The datasets presented in this study can be found in online repositories. The names of the repository/repositories and accession number(s) can be found through the database of Genotypes and Phenotypes (dbGaP; phs001709.v1.p1).
