# Supplementary material for: Genetic insights into the timing of metastasis: a secondary analysis of the Count Me In metastatic breast cancer cohort
Source: Front Cell Dev Biol. 2026 May 29;14:1832248. doi: 10.3389/fcell.2026.1832248 (PMC13260436; doi:10.3389/fcell.2026.1832248)
Supplement: Supplementary file 3 [file Table1.docx]

Supplementary Table 1. Candidate Gene List Used to Generate BED File for Variant Filtering.

| **Gene** | **Chr** | **Start** | **End** |
| --- | --- | --- | --- |
| AHR | chr7 | 17298652 | 17346147 |
| ATF3 | chr1 | 212565407 | 212620777 |
| ATG16L1 | chr2 | 233251673 | 233295669 |
| AXIN2 | chr17 | 65528563 | 65561648 |
| BAX | chr19 | 48954875 | 48961798 |
| BBC3 | chr19 | 47220824 | 47232860 |
| BCHE | chr3 | 165772904 | 165837423 |
| CASC16 | chr16 | 52552087 | 52606975 |
| CCL4 | chr17 | 36103827 | 36105614 |
| CDKN1A | chr6 | 36676463 | 36687332 |
| CHI3L1 | chr1 | 203178931 | 203186704 |
| CTLA4 | chr2 | 203867771 | 203873965 |
| CXCL12 | chr10 | 44370165 | 44385097 |
| CYP1B1 | chr2 | 38067509 | 38076151 |
| DAAM1 | chr14 | 59188667 | 59371405 |
| ERBB4 | chr2 | 211375717 | 212538802 |
| ESR1 | chr6 | 151656672 | 152129619 |
| ESR2 | chr14 | 64226707 | 64338613 |
| GALNT16 | chr14 | 69259631 | 69386337 |
| GAPDH | chr12 | 6534517 | 6538371 |
| HIF1A | chr14 | 61695513 | 61748258 |
| HMGB1 | chr13 | 30456704 | 30617597 |
| IL10 | chr1 | 206767602 | 206772494 |
| IL18 | chr11 | 112143260 | 112164094 |
| IL2RB | chr22 | 37125838 | 37175118 |
| IL6 | chr7 | 22727200 | 22731998 |
| IL7R | chr5 | 35856891 | 35879603 |
| ITCH | chr20 | 34363273 | 34511773 |
| KRAS | chr12 | 25205246 | 25250929 |
| MAP3K21 | chr1 | 233327724 | 233385148 |
| MGMT | chr10 | 129467241 | 129770983 |
| MMP2 | chr16 | 55478830 | 55506691 |
| MMP9 | chr20 | 46008908 | 46016561 |
| NBN | chr8 | 89933331 | 89984667 |
| NFKB1 | chr4 | 102501359 | 102617302 |
| NME1 | chr17 | 51153559 | 51162168 |
| NR5A2 | chr1 | 200027710 | 200177415 |
| **Gene** | **Chr** | **Start** | **End** |
| POLG | chr15 | 89316320 | 89334824 |
| SENP2 | chr3 | 185586295 | 185633551 |
| SOD1 | chr21 | 31659693 | 31668931 |
| SOD2 | chr6 | 159669069 | 159762281 |
| TCF3 | chr19 | 1609292 | 1652615 |
| TFAM | chr10 | 58385410 | 58399220 |
| TGFB1 | chr19 | 41330323 | 41353922 |
| TIMP2 | chr17 | 78852977 | 78925387 |
| TNFRSF11B | chr8 | 118923557 | 118951885 |
| TNFSF11 | chr13 | 42562736 | 42608013 |
| TXNRD2 | chr22 | 19875522 | 19941818 |
| VDR | chr12 | 47841537 | 47904994 |
| VEGFA | chr6 | 43770211 | 43786487 |
